# Digital Media-based Health Intervention on the promotion of Women’s physical activity: a quasi-experimental study

**DOI:** 10.1186/s12889-018-5025-5

**Published:** 2018-01-15

**Authors:** Nooshin Peyman, Majid Rezai-Rad, Hadi Tehrani, Mahdi Gholian-Aval, Mohammad Vahedian-Shahroodi, Hamid Heidarian Miri

**Affiliations:** 1 Social Determinants of Health Research Center, Mashhad University of Medical Sciences, Mashhad, Iran; 2 Health Care Management, Visiting Professor of Faculty of Management, Islamic Azad University, South Tehran Branch, Tehran, Iran

**Keywords:** Health behavior, Health promotion, Physical activity, Women, Health education, Fitness, International physical activity questionnaire (IPAQ)

## Abstract

**Background:**

Technological advances have caused poor mobility and lower physical activity among humankind. This study was conducted to assess the impact of a digital media-based (multi-media, internet, and mobile phone) health intervention on promotion of women’s physical activity.

**Methods:**

In this quasi-experimental study, 360 women were divided into case and control groups. The digital media-based educational intervention was conducted in two months in the case group electronically, using mail and Internet and telephone platforms. Physical activity was measured using International Physical Activity Questionnaire (IPAQ) that estimated women’s physical activity rate in the previous week. Data was analyzed using descriptive and analytical statistics (ANOVA, chi-square, paired and independent t-tests) using SPSS 20.

**Results:**

The mean score of knowledge, attitude and level of physical activity in the control group were not significantly different before and after the intervention. While in the case group, this difference before and after the intervention was significant (*p* < 0.001), and mean scores of the above-mentioned factors increased after the intervention.

**Conclusions:**

Using innovative and digital media-based health education can be effective in improving health-based behavior such as physical activity. Therefore, it seems necessary to develop user-based strategies and strengthen the behavioral change theories and hypotheses based on digital media for effective influence on behavior.

**Trial registration:**

Iranian Registry of Clinical Trials (IRCT), IRCT20160619028529N5. Registered December 24, 2017 [retrospectively registered].

## Background

For various reasons, nowadays people are having more sedentary lifestyle. Physical activity associated with work, home, and transportation has been decreased due to technological advancements and social changes [1]. Increased working hours has led to having less time performing physical activity Paying less attention to a more natural life and spread of urbanization have also attributed to reduced physical activity [2]. Ekelund et al. in 2016 showed that high levels of moderate intensity physical activity (i.e., about 60–75 min per day) eliminates the increased mortality risks associated with high total sitting time [3].

Physical activity is a viable strategy that can affect non-communicable diseases. In 2005, 35 million deaths occurred due to non-communicable chronic diseases (NCDs) worldwide, comprising 60% of all deaths and 47% of the diseases [4]. According to a World Health Organization (WHO) report, these figures will reach 73% (3/4 of all deaths) and 60% of the burden of diseases by 2020. This is even more serious in middle and low-income countries, and has turned into an epidemic that involves about 80% of deaths [5]. Ding et al. in 2016 showed that “low-income and middle-income countries share the largest disease burden from physical inactivity” [6].

The prevalence of obesity among women has been increased. This increase was due to the changes in lifestyles, increasing mechanization of tasks and dramatic decrease in physical activity. Benefits and impact of regular physical activity in promoting health are well-recognized; therefore, interventions should be designed to encourage women to adopt, maintain and enhance such health behaviors [7].

Self-care behaviors, especially during physical activity, are recognized as important factors in maintaining an active lifestyle. Various studies have demonstrated that self-care behaviors are associated with promotion of health, increased physical activity, and weight loss [8].

A possible way to enhance self-care behaviors and increase participants’ involvement in health-related behaviors is the use of digital media, such as the Internet, mobile phone technology, etc. [9]. Thus, in an attempt to increase current rates of physical activity in women, digital media can be used as an effective mechanism [10]. These digital media are promising because of potential for scalability, low cost, use in multiple settings including middle- and low-income nations, and opportunities for real-time modifications and improvements [9].

According to statistics provided by the International Telecommunication Union (ITU) in 2001, there were more than 20,000 health-related sites, and Internet users were estimated as 500 million people. In 2013, this union estimated Internet users as 2.3 billion, which is more than one-thirds of the world’s population [11]. This rate was also estimated to be 3.2 billion Internet users in 2015 [12]. Accordingly, health education and promotion researchers throughout the world are currently trying to explore innovative ways based on the internet and other digital media to increase the efficacy of their interventions [13]. The study of Vollum in 2014 [14] showed that social interaction can be increased and enhanced by the use of social media in educational settings. Vollum’s study [14] also showed that social media have already been using current programs of health/wellness outside the K-12 system.

These media have some advantages including: availability at any time or place, providing efficient content and appropriate interaction with the user. Therefore, these media provide a good platform for people to monitor their health behaviors such as monitoring the level of physical activity to improve health [15]. Therefore, it seems that digital media-based intervention can increase physics activity.

Thus, given the highly important role of regular physical activity in improving women’s life quality, and valuable women’s role in forming an active family and social lifestyle, the present study was conducted to assess the impact of digital media-based intervention (multi-media, internet, and mobile phone) to promote women’s physical activity.

## Methods

### Design

This was a quasi-experimental study. Its population was women who referred to health centers in Kerman, Iran. At the time of the study, there were 8 active health centers in Kerman, in which 4 were randomly selected as intervention centers and 4 as control ones.

### Sample

According to the following formula, considering type 1 error of 0.05 and power of 80%, and assuming 15% score difference before and after the intervention, 180 women were calculated as the sample size, and the same number as the control group. Therefore, 360 women were recruited.

$$ \frac{{\left[z\left(1-\frac{\alpha }{2}\right)\sqrt{2p\left(1-p\right)}+z\Big(1-\beta \sqrt{p0\left(1-p0\right)+p1\left(1-p1\right)}\right]}^2}{{\left(p1-p0\right)}^2}\times D $$

**Fig. 1:**
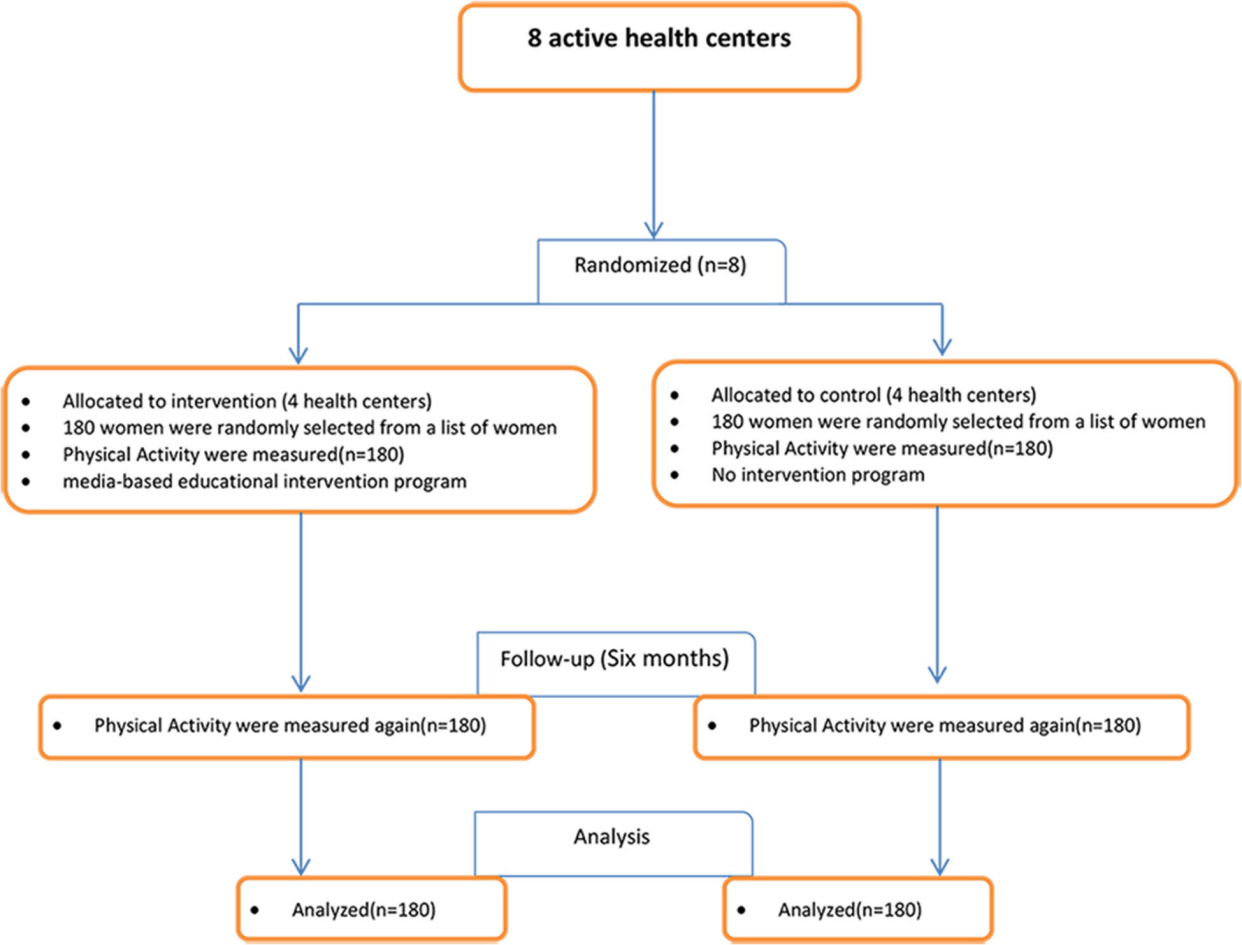
Flow chart of the study

The members of the intervention group were selected from 4 health centers in Kerman. The samples were randomly selected from a list of women visiting the health centers and then the intervention was performed. Inclusion criteria were: being older than 18 years, having a mobile phone and the ability to use it, access to the internet, ability to use a computer and the internet, and willingness to participate. The invitation to participate in the study was sent electronically and/or by mobile to those who had the conditions to participate in the study. Given that centers were governmental, all people participated in the study willingly (Fig. 1).

### Measures

Body mass index (BMI) was calculated using the respondents’ reported height and weight. Physical activity was measured using the International Physical Activity Questionnaire (IPAQ) which estimates women’s physical activity rate in regarding MET-minutes/week [16]. A separate questionnaire was used to investigate the knowledge and attitude. This questionnaire consisted of 12 questions. Six multiple choice questions were used to measure knowledge and six five-point Likert style (Completely agree, no idea, disagree, completely disagree) questions were used to measure attitudes. After preparing the knowledge and attitude questionnaire, content validity and face validity were performed to test the reliability of the instrument. A literature review was performed and experts and professors related to the subject of the study were consulted to edit the questionnaire. When the face validity of the instrument was verified, by using the content validity index (CVI) and content validity ratio (CVR), the questionnaire was sent to 10 experts in public health and health information technology; their comments and view were used to eliminate possible defects of the questionnaire. To test the validity of the questionnaire, Cronbach’s alpha coefficient was used. The questionnaire was filled-out by 20 women referring to health centers in Kerman, Iran and was filled-out again after 2 weeks. The Cronbach’s alpha coefficient of internal consistency of the questionnaire was estimated between 82% and 88% and its interclass correlation coefficient was 71–77%.

### Analysis

Data was then analyzed using descriptive (frequency distribution, mean and standard deviation) and analytical statistics such as ANOVA, paired and independent t-tests by SPSS_20_. A paired *t*-test was conducted to compare changes before and after the intervention. Two groups comparison was performed using Independent sample t-test.

### Intervention

The unique characteristic of this study was its digital media-based electronic educational intervention. In the first step, the research team explained the importance and the objectives of the study to attract women’s cooperation. Moreover, women were briefed on how to answer the questions using a completed typical questionnaire, and further explanations were also first given if needed. Data was collected at the first stage (pre-test), simultaneously, in all 8 centers.

When the pretest was completed, media-based educational intervention program was performed for the intervention group. Virtual space in this study was used in the following way. An educational website related to physical activity was designed with different sections. Besides different educational stuff related to physical activity, the advantages of such activities and regular exercises and educational films were also included. In addition to watch films on this site, women could download the films on regular mobile phones and use it. The site also had an electronic section in which women could assess their physical activity and BMI online and therefore, they were encouraged to self-monitor their physical activity. Due to the Iranian culture and religious beliefs of Muslim women and therefore restrictions of physical activity for women in every place, a section of the site was assigned to introduce suitable women-only places devoted to physical activity with photos and its characteristics, such as women-only parks, female-only sessions, female-only gyms and sport complexes, etc. To that end, an educational CD with several chapters was prepared in an auto-run format according to the latest physical activity topics by the Ministry of Health and Medical Education of Islamic Republic of Iran. This CD was available for women participating in the study. Furthermore, an educational website on women’s physical activity was designed with different sections. In addition to various trainings about physical activity and the benefits of regular exercise in the website, a page was designed for educational video clips, enabling women to watch videos, or download them on their mobiles. In the chat room section, participants could exchange information.

Mobile phone numbers of participants and people whose views had influence on the participants’ physical activity (i.e. supporters) were also registered in the pretest. Then, SMS messages about the importance of physical activity were sent daily to supporters and participants. Six months after intervention, the impact of interventions was assessed and data collected using the same tools.

## Results

The demographic and socioeconomic characteristics of the women are presented in Table 1. The two groups were not significantly different regarding demographic characteristics except for weight (Table 1).

**Table 1:** Frequency Distribution of Personal Details among Control and Case Groups

Variable	Control group	Case group	*p*
Marital status			df = 1 p** = 0.147
*Single*	68(37.2%)	54(30%)	
*Married*	113(62.8%)	126(70%)	
Education level			df = 3 p** = 0.638
*High school*	68(37.8%)	58(32.2%)	
*Associate Degree*	17(9.4%)	22(12.2%)	
*BSc.*	77(42.8%)	79(43.9%)	
*MSc. & above*	18(10%)	21(11.7%)	
Age	31.933 ± 9.458	33.411 ± 9.010	p* = 0.130
Height (cm)	161.39 ± 7.488	162.38 ± 5.738	p* = 0.159
Weight (kg)	63.022 ± 12.366	67.288 ± 11.958	p* < 0.001
Body Mass Index (BMI)	24.353 ± 5.576	25.522 ± 4.370	p* < 0.05

** ^Pearson Chi-Square;^ * ^Independent t test^

Before the intervention, women’s mean weight was 63.022 kg in the control group and 67.288 kg in the case group; there was a significant difference between the two groups (*p* < 0.05). However, after the intervention, mean weight decreased to 66.388 kg in the case group, but increased to 63.07 kg in the control group (*p* < 0.001). Before the intervention, no significant difference was found regarding other demographic characteristics.

Before the intervention, BMI was 24.353 kg/m^2^ in the control group and 25.522 kg/ in the case group; the difference between the two groups was significant (*p* = 0.028). After the intervention, BMI increased to 24.404 kg/m^2^ in the control group, but the increase was not significant (*p* = 0.664) based on paired t-test. In the case group, mean BMI decreased to 25.186 kg/m^2^, which was statistically significant based on paired t-test (*p* < 0.001).

The results showed no significant differences between the two groups in terms of knowledge, attitude, and physical activity before the intervention. However, according to independent t-test, the difference between the two groups was significant after the intervention (*p* < 0.05), and mean scores of the above- mentioned scales increased among the intervention group (Table 2).

**Table 2:** Mean and Standard Deviation of Women’s Knowledge, Attitude, and Physical Activity among Control and Case Groups before and after Intervention

Variable	Mean and Standard Deviation Before Intervention			Mean and Standard Deviation After Intervention		
	*Control group*	*Case group*	*p-value*	*Control group*	*Case group*	*p-value*
Knowledge	0.8156 (±0.0600)	0.9611 (±0.641)	p* = 0.099	0.8636 (±0.0744)	6.116 (±0.0600)	p* < 0.000
Attitude	16.15 (±2.54)	16.16 (±3.412)	p* = 0.136	16.70 (±2.87)	21.10 (±2.747)	p* < 0.001
Physical activity (MET/min)	838.44 (±96.781)	992.17 (±83.302)	p* = 0.229	881.82 (±90.482)	3604.32 (±271.195)	p* < 0.001

*Independent t test

**Fig. 2:**
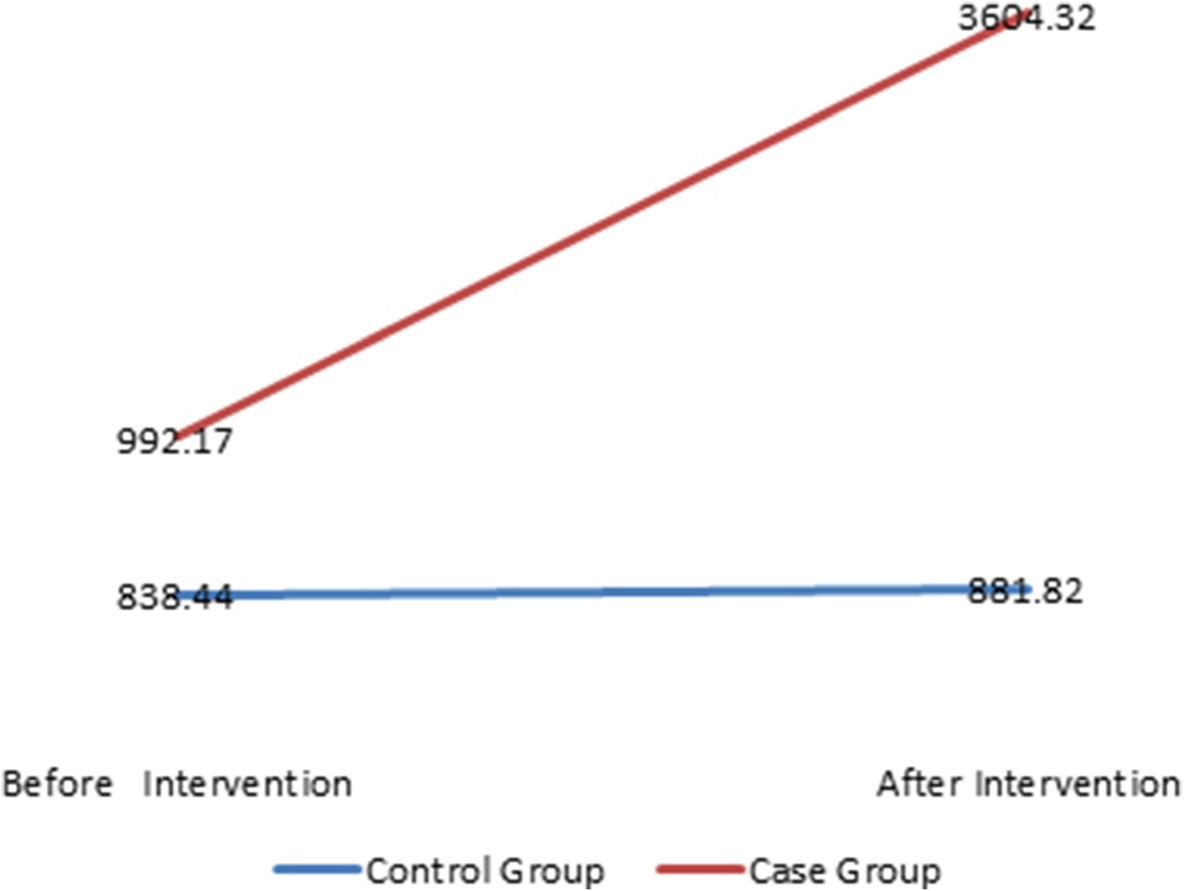
Mean of Women’s Physical Activity before and after Intervention in Control and Case Groups

The mean score of knowledge, attitude and level of physical activity in the control group was not significantly different before and after the intervention (Table 3). While in the case group, this difference before and after intervention was significant (*p* < 0.001), and mean scores of the above-mentioned scales increased after the intervention (Fig. 2).

**Table 3:** Mean and Standard Deviation of Women’s Knowledge, Attitude, and Physical Activity before and after Intervention among each of the Control and Case Groups

Variable	Control Group SD ± M			Case Group SD ± M		
	*Before Intervention*	*After Intervention*	p*	*Before Intervention*	*After Intervention*	p*
Knowledge	0.816 (±0.0600)	0.8636 (± 0.0744)	p* = 0.586	0.9611 (±0.641)	6.116 (±0.0600)	p* < 0.001
Attitude	16.65 (± 2.54)	16.70 (± 2.87)	p* = 0.859	16.15 (± 3.41)	21.10 (± 2.74)	p* < 0.001
Physical activity	838.44 (± 96.781)	881.82 (± 90.482)	p* = 0.566	992.17 (± 83.302)	3604.32 (± 271.195)	p* < 0.001

*Paired t test

Table 4 indicates that the effects of the intervention, time and the interaction of time and intervention are statistically significant for knowledge, attitude and physical activity. However, as it is illustrated in Table 3 the score of knowledge, attitude and level of physical activity are not considerably changed over two measurements among controls group. This could be explained by the significant effect of interaction between time and intervention that emphasizes the effect of time differs noticeably between cases and controls.

**Table 4:** Results from repeated measure analysis of variance for knowledge, attitude physical activity

	Knowledge			Attitude			Physical Activity		
Source	F	P-value	Adjusted R-squared	F	P-value	Adjusted R-squared	F	P-value	Adjusted R-squared
Model	10.44	0.0000	0.8266	2.44	0.0000	0.4222	2.91	0.0000	0.4893
intervention	864.49	0.0000		68.19	0.0000		47.91	0.0000	
time	1071.5	0.0000		144.59	0.0000		94.52	0.0000	
intervention × time	1034.37	0.0000		150.19	0.0000		85.13	0.0000	

## Discussion

This study was conducted to assess the impact of digital media-based (multimedia, internet, and mobile phone) intervention and education on women’s physical activity. Use of cyberspace and digital technology in a specific group (Muslim women) was one of the most prominent features of this study compared to other studies conducted in this area especially in Iran.

Generally, before the intervention, control and case groups were similar regarding their demographic characteristics. The results showed a significant increase of physical activity of women who used educational multimedia and websites and received daily text messages, compared to those in the control group. This indicates a positive impact of media, as educational interventions, on health promotion.

Ornes and Ransdell [17] also investigated the effect of web-based education on female university students’ physical activity who received education through internet for 4 weeks. By the end of the study, mean number of steps per day had increased by 38.8% among the group that received education through the internet; which indicates the positive effect of using internet and web on physical activity. The result of this study concurs with the results of Ornes and Ransdell’s investigation.

With rapid growth of digital media, including internet and mobile phones, and increasing number of people with access to such media use of these media seems necessary to encourage physical activity [18]. The strength of these media, compared to print media and face-to-face intervention, is educating large numbers of people at low costs [19].

In the present study, in addition to a significant increase in physical activity in the case group, women’s knowledge and attitude also significantly increased after the intervention; while no increase was observed in the control group. This shows that digital media, such as internet, computer software, and mobile phone-based programs, encourage women to perform physical activity, and increase their knowledge and attitude.

Knowledge, attitude and behavior in our daily lives are intertwined with each other. Changes in knowledge cause changes in attitude. Change in attitude also causes changes in behavior. Educational programs with using digital media, such as internet, computer software, and mobile phone-based ones are effective in improving knowledge and attitudes to more physical activity [17] .

Palmer et al. [20] also conducted a web-based intervention among students. Palmer et al. study showed students’ increased attitude and knowledge about physical activity, and indicated the positive effect of internet-based intervention on their knowledge and attitude.

Knowledge of the risks and benefits of lifestyle-associated behaviors are the prerequisites of performing a behavior. If people lack the relevant knowledge, they will not accept reasons for enduring difficulties associated with that behavior [21]. Digital media are capable of providing information in the shortest possible time with maximum efficiency, and have a huge impact on knowledge, literacy, and attitudes of their audience [22].

Another important result of the present study was about BMI variations before and after the intervention. Although two groups’ BMI showed a significant difference before and after the intervention, BMI had a significant drop in the case group, after the interventions, and a slight increase in the control group. Thus, digital media caused increasing physical activity, which decreased in weight and an improvement in BMI in the case group.

In a study performed by Cavallo et al. [23] to assess the effect of social media-based intervention on physical activity, physical activity and social support increased in the case group and 66% of the participants recommended the use of digital media-based programs to their friends. Bennett et al. [24] also showed that internet-based weight loss interventions can be useful. In their study, they used interventions based on the internet and mobile phone for 12 weeks, which led to weight loss in the group that received education through the internet and mobile phone. Furthermore, weight loss was more prominent in participants who used more internet interventions.

### Strengths and limitations

Strengths of this study were using virtual space and mobile phones for promotion of physical activity that provided the space for exchanging ideas and suggestions by participants. Limitations of the present study were low speed and narrow internet bandwidth and, sometimes, internet disconnection. Another limitation of this study was that the study was conducted only among women, thus findings from this study cannot be generalized to the general population. In addition, given that data collection was based on self-reporting there was the possibility of bias in a more favorable response and moreover, physical activity was not objectively assessed via a physical activity assessment or activity trackers.

## Conclusion

It seems that media-based interventions (multimedia, internet, and mobile phone) can positively affect physical activity of women. Therefore, it seems essential to develop user-based strategies and strengthen behavioral change theories and hypotheses based on digital media by developing virtual and digital media use in the community.

## Abbreviations

BMI: Body mass index
IPAQ: International Physical Activity Questionnaire
